# A multiplex PCR–Cas12a–lateral flow platform for pooled screening of four major tobacco pathogen groups

**DOI:** 10.3389/fpls.2026.1919480

**Published:** 2026-08-04

**Authors:** Yena Zhang, Changwen Ye, Bo Zeng, Hongru Xi, Baojian Wu, Kuo Huang, Dandan Liu, Guokang Chen, Chenqi Niu

**Affiliations:** 1 China Tobacco Standardization Research Center, Zhengzhou Tobacco Research Institute of CNTC, Zhengzhou, China; 2 College of Plant Protection, Southwest University, Chongqing, China

**Keywords:** fluorescent detection, lateral flow dipsticks, molecular diagnostics, multiplex PCR–Cas12a, tobacco pathogens

## Abstract

Tobacco production is increasingly threatened by pathogens such as *Ralstonia*, *Phytophthora*, *Alternaria*, and *Pseudomonas*, which cause bacterial wilt, black shank, brown spot, and wildfire, respectively. Conventional diagnostics typically target single pathogens, and reliable methods for simultaneous multi-pathogen detection remain limited. Here, we developed a multiplex PCR–Cas12a–LFD platform for the pooled multi-target screening of four major tobacco pathogens. Multiplex PCR amplifies pathogen-specific 16S rRNA and ITS regions, while Cas12a–crRNA complexes specifically recognize the amplicons and trigger trans-cleavage of fluorophore–quencher or fluorophore–biotin probes, enabling fluorescence and lateral flow readouts. Reaction parameters were systematically optimized to reduce primer interference and achieve balanced amplification across targets. The individual target groups showed different mass-based analytical LODs, ranging from 5 fg/μL to 5 pg/μL under the tested conditions. For an equal-proportion mixed-template series, the universal reporter produced a pooled-signal LOD of 186 fg/μL and showed no detectable signal from the tested non-target DNA panel. LFD-based visual detection reached 0.5 pg/μL, while preliminary spiked-leaf tests at two extracted-DNA levels yielded apparent recovery rates of 93%–95%, indicating matrix compatibility under the tested conditions. This platform provides a laboratory-based pooled screening tool for preliminary sample triage, with target-specific identification requiring subsequent confirmatory analysis.

## 1 Introduction

Tobacco (*Nicotiana tabacum*) is a globally important economic crop (Lewis, 2020; Martins-Da-Silva et al., 2022). However, in recent years, the incidence and severity of tobacco diseases have increased markedly due to climatic and environmental changes, severely affecting leaf development and leading to substantial economic losses (Ali et al., 2023; Cheeseman, 2024; Khan et al., 2024; Ziska and Parks, 2024).

The major diseases affecting tobacco include wildfire, black shank, bacterial wilt, and brown spot. Tobacco bacterial wilt, caused by *Ralstonia solanacearum*, is considered one of the most destructive bacterial plant diseases worldwide, with yield losses exceeding 50% in certain production areas (Cai et al., 2021; Ahmed et al., 2022). Tobacco black shank, a severe soil-borne disease caused by *Phytophthora nicotianae*, has exhibited a continuously expanding distribution since its initial identification (Gai et al., 2023; Pandey, 2023; Cochran et al., 2024). Brown spot, caused by *Alternaria alternata*, is regarded as the second most damaging disease in tobacco-growing regions (Xie et al., 2021; Shuo et al., 2023; Garmendia et al., 2025). Wildfire disease, induced by *Pseudomonas syringae* pv. *tabaci*, has emerged as one of the most prevalent bacterial infections in major tobacco-producing areas (Liu et al., 2021; Li et al., 2025a).

These representative and frequently occurring diseases severely compromise tobacco leaf quality, reduce both yield and grade of flue-cured tobacco, and result in substantial economic losses. Without timely detection and effective control, the pathogens can spread rapidly, leading to large-scale epidemics, yield decline, and deterioration of product quality. Therefore, the rapid and accurate preliminary detection of tobacco pathogens is essential for effective disease management and prevention.

Currently, the gold standard for diagnosing these diseases relies on the design and synthesis of specific primers, followed by polymerase chain reaction (PCR) for rapid and accurate identification of pathogens (Lydon and Patterson, 2001; Huang et al., 2009; Meng and Wang, 2010; Long et al., 2021). With advances in molecular biology, isothermal amplification–based assays have also been developed for the detection of representative plant pathogens (Le and Vu, 2017; Panno et al., 2020; Zou et al., 2020; Ivanov et al., 2021). Moreover, the integration of DNA-based detection methods with lateral flow dipsticks (LFDs) allows for intuitive, on-site visualization of results (Dai et al., 2019; Xu et al., 2023). In particular, LFD-based assays for bacterial wilt have been reported to markedly improve detection efficiency and sensitivity in tobacco disease diagnostics (Li et al., 2021).

However, in agricultural production, multiple tobacco diseases frequently occur concurrently, while conventional single-pathogen detection strategies remain inadequate for efficient and comprehensive diagnosis (Fang and Ramasamy, 2015). With the rapid advancement of artificial intelligence (AI), image recognition–based approaches for multiplex monitoring of tobacco diseases have been developed in recent years. For instance, Shao et al. (2017) established a diagnostic method that integrates multi-feature extraction and genetic algorithm–optimized backpropagation (BP) neural networks to identify tobacco diseases, whereas Lin et al. (2022) proposed a convolutional neural network (CNN) model, CAMFFNet (Coordinate Attention–Based Multiple Feature Fusion Network), for field-level image-based identification of tobacco diseases. Despite these developments, more precise multiplex detection methods that directly target the genetic material of tobacco pathogens remain limited.

The groundbreaking discovery of Clustered Regularly Interspaced Short Palindromic Repeats (CRISPR) and their associated proteins has positioned the CRISPR/Cas system at the forefront of modern nucleic acid detection technologies, owing to its operational simplicity, remarkable sensitivity, and unparalleled specificity (Aman et al., 2020; Kim et al., 2021; Dronina et al., 2022). Among the Cas effectors, Cas12a functions as an RNA-guided endonuclease that targets double-stranded DNA (dsDNA). Upon recognition of its specific DNA target, Cas12a undergoes conformational activation and subsequently exhibits nonspecific cleavage activity toward single-stranded DNA (ssDNA) reporters. *In vitro*, Cas12a can be programmed with a CRISPR RNA (crRNA) to form a binary Cas12a–crRNA complex that specifically identifies target dsDNA sequences. Once activated, Cas12a triggers collateral (*trans-*) cleavage of other ssDNA molecules present in the reaction system (Li et al., 2018). Fluorescence-based reporting assays that exploit this trans-cleavage property of Cas12a have been successfully applied to a wide range of DNA detection platforms (Wang et al., 2019; Williams et al., 2019; Yin et al., 2021; Mao et al., 2022; Yang et al., 2024). While one-pot RPA-Cas or LAMP-Cas systems are excellent for point-of-care, single-target diagnostics, they are notoriously difficult to multiplex. Attempting to amplify four discrete targets simultaneously in a single isothermal reaction typically leads to severe non-specific primer interactions and chaotic amplification (Wang et al., 2020; Liu et al., 2022, 2024; Pataer et al., 2024; Bao et al., 2025; Wang et al., 2025b).

In our previous study, we established separate PCR–Cas12a–LFD assays for detecting the same four tobacco-associated target groups and demonstrated the feasibility of combining target-specific PCR amplification with Cas12a-mediated lateral-flow readout (Niu et al., 2026). However, those assays were operated as separate target-specific detection configurations and did not provide a pooled workflow for screening the four target groups in a single multiplex PCR. The present study builds directly on that foundation by integrating the four primer pairs into one multiplex amplification reaction and the corresponding crRNAs into a pooled Cas12a detection reaction. Thus, the methodological objective of the current work was to optimize amplification and recognition balance in a pooled multi-target screening format rather than to re-establish the individual target-specific assays.

Building on our previous target-specific PCR–Cas12a–LFD assays, we developed a pooled multiplex PCR–Cas12a platform for screening four tobacco-associated target groups: *Pseudomonas*, *Phytophthora*, *Ralstonia*, and *Alternaria*. Four primer pairs were combined in one multiplex PCR, followed by Cas12a recognition using four corresponding crRNAs and a universal fluorescence or LFD reporter. A positive signal indicates that at least one target group is present but does not identify the individual target responsible. The platform is therefore intended for preliminary laboratory screening, with pathogen-resolved identification requiring subsequent target-specific analysis.

## 2 Materials and methods

### 2.1 Materials

The microbial strains used in this study were laboratory-preserved strains, including *R. solanacearum*, *P. syringae* pv. *Tabaci*, *A. alternata*, and *P. nicotianae*. Plasmids carrying the target DNA (NR_044040.1, AH015112.2, OL958426.1, and NR_043716.1) of various strains were synthesized by BGI (Beijing Genomics Institute, China). Instruments: LightCycler 480 II real-time PCR system (Roche, USA), Varioskan LUX microplate reader (Thermo Scientific, USA), TC-96/G/H(b)B thermal cycler (Bioer Technology, China), SINSAGE-EP300 electrophoresis system (Sensage, China). Reagents: 2×TaqMasterMix (NEB, USA), EnGen Lba Cas12a (Cpf1) (NEB, USA), 10×NEBuffer™2.1 (NEB, USA) (1× Buffer Components: 50 mM NaCl, 10 mM Tris-HCl, 10 mM MgCl_2_, 100 µg/ml BSA, pH 7.9@ 25 °C), and Cas12a nucleic acid detection LFD (Tiosbio, China). The genome extraction kit was purchased from Solarbio (Beijing, China). All oligonucleotide sequences used in this study were synthesized by BGI (Beijing Genomics Institute, China).

### 2.2 Pathogen culture conditions and genome extraction

Single colonies of *Ralstonia solanacearum* and *Pseudomonas syringae pv. tabaci* were first obtained by streaking on LB agar plates. These colonies were then transferred into LB broth and cultivated overnight at 37 °C with agitation at 180 rpm to generate bacterial suspensions for subsequent DNA extraction. For the fungal pathogen *Alternaria alternata*, isolates grown on PDA agar were inoculated into liquid fungal medium and incubated at 28 °C with shaking at 180 rpm for 48 h. Similarly, the oomycete *Phytophthora nicotianae* was cultured from single colonies on V8 agar, followed by growth in V8 broth under the same conditions (28 °C, 180 rpm, 2 days) to obtain mycelial suspensions for DNA preparation. Genome extraction was performed according to the supplier’s instructions.

### 2.3 Primer and crRNA design and in silico sequence evaluation

Primer pairs were selected from the 16S rRNA regions of the bacterial target groups and the ITS regions of the fungal and oomycete target groups. Candidate amplicons were required to contain an LbaCas12a-compatible PAM sequence (TTTN) adjacent to the selected crRNA protospacer. Each full-length crRNA consisted of the LbaCas12a direct-repeat sequence followed by a 20-nt target-specific spacer. The primers and crRNAs were designed to support broad detection of the selected taxonomic groups rather than differentiation of pathogenic and non-pathogenic strains. The complete sequences and characteristics of all primers, crRNAs, and ssDNA reporter probes are provided in **Supplementary Table 1**.

The primer pairs were evaluated against the core_nt database using NCBI blast tool. The analysis summarized in **Supplementary Table 2** was intended to estimate taxonomic inclusivity within selected target taxa, whereas **Supplementary Table 3** provides a limited in silico comparison against three selected non-target genera. The reported counts represent database sequence records meeting the specified matching criteria, not unique strains or experimentally verified pathogenic isolates. Because pathogenicity cannot generally be inferred from conserved 16S rRNA or ITS sequences, this analysis was not used to claim pathogenic-strain specificity.

In the complete assay, a detectable signal requires both PCR amplification and recognition of a PAM-adjacent protospacer by the corresponding Cas12a–crRNA complex. This dual-recognition design provides an additional sequence-dependent filter against nonspecific PCR products. Nevertheless, a non-pathogenic or closely related organism carrying both the required primer-binding sites and the Cas12a target sequence may still generate a positive signal. Accordingly, the assay result is interpreted at the target-group level.

### 2.4 Singleplex PCR–Cas12a reaction

The 20 μL PCR amplification system consisted of 10 μL of 2× Taq Master Mix, 0.8 μL each of forward and reverse primers (10 μM), 1 μL of template DNA, and ddH_2_O added to a final volume of 20 μL. The PCR amplification program was as follows: initial denaturation at 94 °C for 2 min; 30 cycles of 94 °C for 30 s, 58 °C for 30 s, and 72 °C for 1 min; followed by a final extension at 72 °C for 5 min.

For Cas12a detection, a 100 μL reaction system was prepared in a 96-well plate, containing 10 μL of PCR product as the activator, 10 μL of 10× Buffer 2.1, 10 μL of F–Q ssDNA probe (10 μM), 3 μL of crRNA (1 μM), 3 μL of Cas12a (1 μM), and ddH_2_O added to a total volume of 100 μL. The fluorescence signal was monitored at 37 °C for 20 min using a microplate reader, with readings taken every 1 min. The excitation wavelength was set at 488 nm and the emission wavelength at 520 nm.

### 2.5 Multiplex PCR–Cas12a reaction

The multiplex PCR amplification was carried out in a total volume of 20 μL, containing 10 μL of 2× Taq Master Mix, mixed primers of *Pseudomonas* sp., *Phytophthora* sp., *Ralstonia* sp., and *Alternaria* sp. at final concentrations of 0.4, 0.1, 0.6, and 0.4 μM, respectively, and 1 μL of each template DNA. The amplification program consisted of an initial denaturation at 94 °C for 2 min; followed by 25 cycles of 94 °C for 30 s, 58 °C for 30 s, and 72 °C for 1 min; and a final extension at 72 °C for 5 min.

For the Cas12a fluorescence detection assay, a total reaction volume of 100 μL was prepared, including 10 μL of multiplex PCR product as the activator, 10 μL of 10× buffer, 10 μL of F–Q ssDNA probe (10 μM), crRNAs (1 μM) for *Pseudomonas* sp., *Phytophthora* sp., *Ralstonia* sp., and *Alternaria* sp. at volumes of 6, 6, 6, and 3 μL, respectively, 21 μL of Cas12a (1 μM), and ddH_2_O to a final volume of 100 μL. Fluorescence was monitored at 37 °C for 20 min using a microplate reader, with data collected every 1 min. The excitation and emission wavelengths were set at 488 nm and 520 nm, respectively.

For lateral flow dipstick (LFD) visualization, a 100 μL Cas12a reaction mixture was similarly prepared using the F–B ssDNA probe (10 μM) in place of the F–Q probe. After incubation at 37 °C for 20 min, an LFD strip was inserted into the reaction mixture, and the results were recorded after 1 min of development.

### 2.6 Spiked samples detection

Fresh, apparently disease-free tobacco leaves were collected from Nanyang and Zhumadian, Henan Province, China. Ten grams of each leaf sample was homogenized, after which cultured materials of *R. solanacearum*, *P. nicotianae*, *A. alternata*, and *P. syringae* pv. *tabaci* were added to the homogenates to prepare simulated mixed-pathogen samples. The bacterial cultures and fungal/oomycete mycelial materials were not independently enumerated by plate counting, a hemocytometer, or another quantitative microbiological method before spiking. Consequently, pathogen burdens expressed as CFU/g, cells/g, spores/g, or propagules/g could not be determined from the current experiment.

The plant tissues and simulated mixed-pathogen samples were rapidly frozen in liquid nitrogen and homogenized into a fine powder using a tissue homogenizer. Total microbial DNA was subsequently extracted using the Microbiome DNA Isolation Kit (EE401) according to the manufacturer’s instructions. The extracted DNA preparations were assessed using a NanoDrop spectrophotometer, and samples with an *A*260​/*A*280​ ratio between 1.8 and 2.0 were used in the assay. Extracted DNA preparations corresponding to the two tested levels, 50 pg/μL and 5 ng/μL, were analyzed using the multiplex PCR–Cas12a assay. Target-equivalent concentrations were estimated from the established fluorescence calibration curve, and apparent recovery rates were calculated to preliminarily assess assay performance in the tobacco-leaf matrix.

This spiking experiment was designed as a preliminary assessment of matrix compatibility at the two tested extracted-DNA levels. It was not designed to determine a pathogen burden–based detection limit in tobacco leaves or to independently evaluate the effect of progressively increasing host DNA concentrations on the detection of low-abundance pathogens.

### 2.7 Data analysis

One-way ANOVA was employed, and values are presented as mean ± standard deviation (n=3). “ns” represents not significant (p>0.05), and “****” represents a highly significant difference (p<0.0001). Fluorescence experiments and lateral flow assays were performed using three independent replicate reactions unless otherwise specified, and values are presented as mean ± standard deviation.

## 3 Results

### 3.1 Principle of the detection method

**Figure 1:**
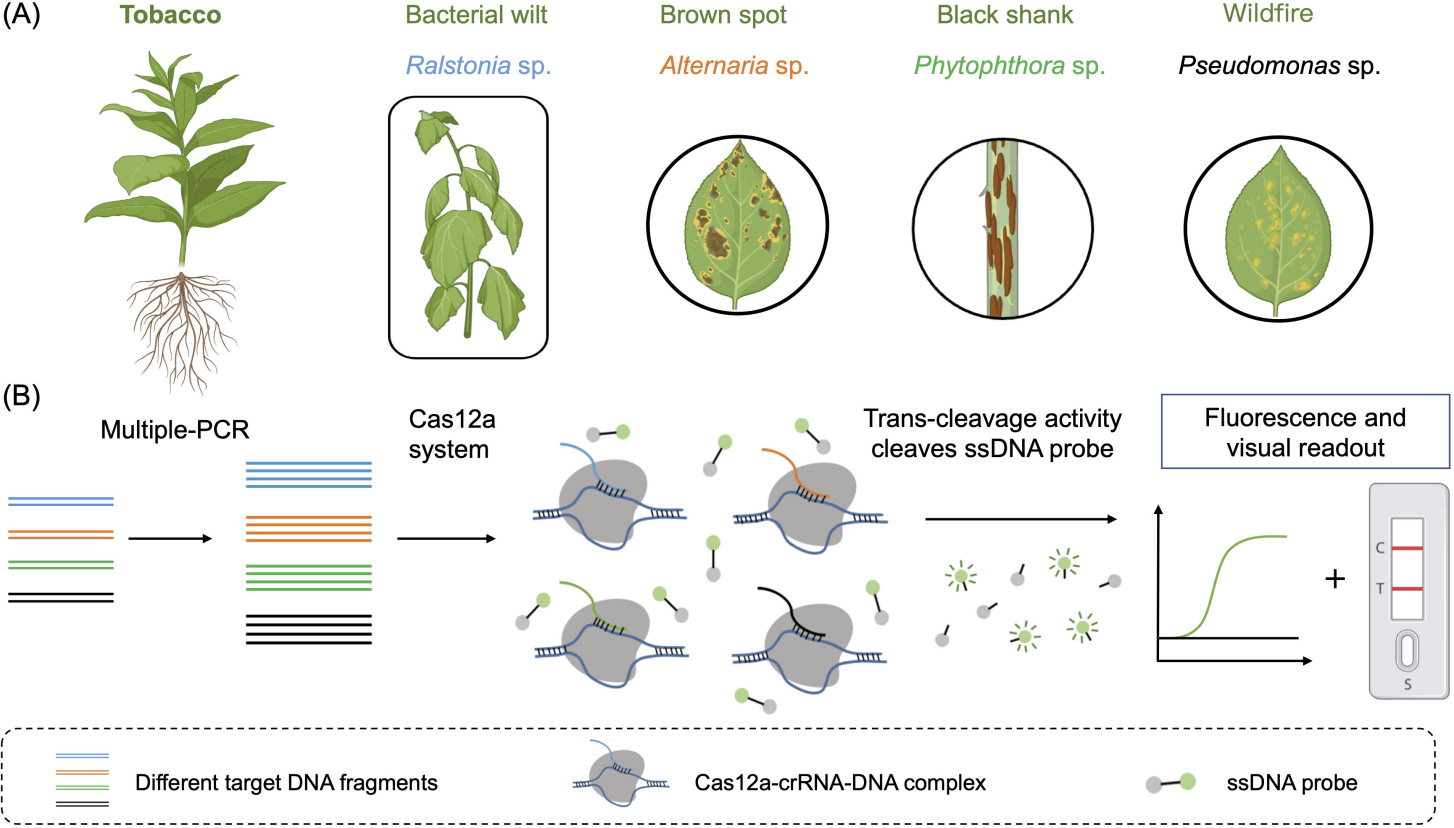
Major tobacco pathogens and schematic illustration of the multiplex PCR–Cas12a-based detection method. **(A)** Representative tobacco diseases—bacterial wilt, black shank, brown spot, and wildfire—and their corresponding pathogens (*Ralstonia* sp., *Phytophthora* sp., *Alternaria* sp., and *Pseudomonas* sp.). The symptom illustrations are intended only to provide visual context and are not used as evidence of pathogen identity. **(B)** Schematic diagram of the detection principle, showing both fluorescence-based detection and LFD-assisted visual detection integrated within the multiplex PCR–Cas12a system.

We established a multiplex detection strategy integrating PCR amplification with the Cas12a system, enabling pooled nucleic-acid screening of *Ralstonia* sp., *Phytophthora* sp., *Alternaria* sp., and *Pseudomonas* sp. for the preliminary screening of tobacco pathogens (**Figure 1A**).

In the presence of target pathogens, the assay initiates with a multiplex PCR step. Specific primer pairs were designed to amplify the 16S rRNA regions of *Ralstonia* sp. and *Pseudomonas* sp., as well as the internal transcribed spacer (ITS) regions of *Phytophthora* sp. and *Alternaria* sp. The primer sequences were carefully optimized to ensure that the resulting amplicons contained the protospacer adjacent motif (PAM) sequence (TTTN) required for Cas12a activation (**Supplementary Table 1**).

Following amplification, the PCR products were incubated with four distinct Cas12a–crRNA complexes, each programmed to recognize a specific sequence downstream of the PAM site within its corresponding amplicon. Upon hybridization between the crRNA and target amplicon, a ternary Cas12a–crRNA–DNA complex is formed, triggering a conformational rearrangement that activates the nuclease’s robust trans-cleavage activity.

Activated Cas12a subsequently cleaves a fluorophore–quencher (F–Q) probe—an ssDNA reporter labeled with a 5′-FAM fluorophore and a 3′-BHQ1 quencher. Cleavage separates the fluorophore from the quencher, generating a measurable fluorescence signal. Alternatively, the F–Q probe can be replaced by a fluorophore–biotin (F–B) probe, an ssDNA reporter labeled with a 5′-FAM and a 3′-biotin group. In the presence of the target, Cas12a activation results in F–B probe cleavage, producing a visible signal on the test line of a LFD for on-site detection. Conversely, in the absence of the target sequence, both F–Q and F–B probes remain intact, yielding no detectable fluorescence or colorimetric signal.

It is important to note that the current single-reporter configuration acts as a universal presence/absence alert for the four target pathogens. A positive LFD signal signifies the potential infection by at least one of these major pathogens, prompting the need for containment or further downstream target-group-level screening if detailed epidemiological profiling is required.

### 3.2 Feasibility analysis

To evaluate the potential taxonomic inclusivity of the primer pairs, an in silico comparison was performed against selected sequence records in the NCBI core_nt database. As summarized in **Supplementary Table 2** and **Supplementary Figures S1**-**S4**, the primer pairs matched numerous database records assigned to the intended target genera or species complexes. These results indicate broad sequence coverage within the selected taxonomic groups. However, the counts represent database records rather than unique strains, and they do not indicate whether the corresponding isolates are pathogenic to tobacco. Thus, the analysis supports target-group inclusivity but does not demonstrate pathogenic-strain specificity.

A limited in silico comparison was also performed against three selected non-target genera commonly associated with plant or soil environments: *Rhizobium*, *Streptomyces*, and *Pectobacterium* (**Supplementary Table 3**). Few or no records meeting the specified primer-matching criterion were identified for most primer pair–non-target genus combinations, although candidate matches were observed for the *Pseudomonas* primer pair in the *Rhizobium* and *Streptomyces* datasets. This limited analysis should not be interpreted as comprehensive evidence of exclusivity against all tobacco-associated microorganisms. Furthermore, because the selected 16S rRNA and ITS regions are not pathogenicity markers, closely related non-pathogenic organisms carrying both the primer-binding sites and the corresponding PAM-adjacent crRNA target sequence could potentially generate a positive result. Accordingly, the platform is intended for broad target-group screening rather than pathogenic-strain or pathovar-level confirmation.

**Figure 2:**
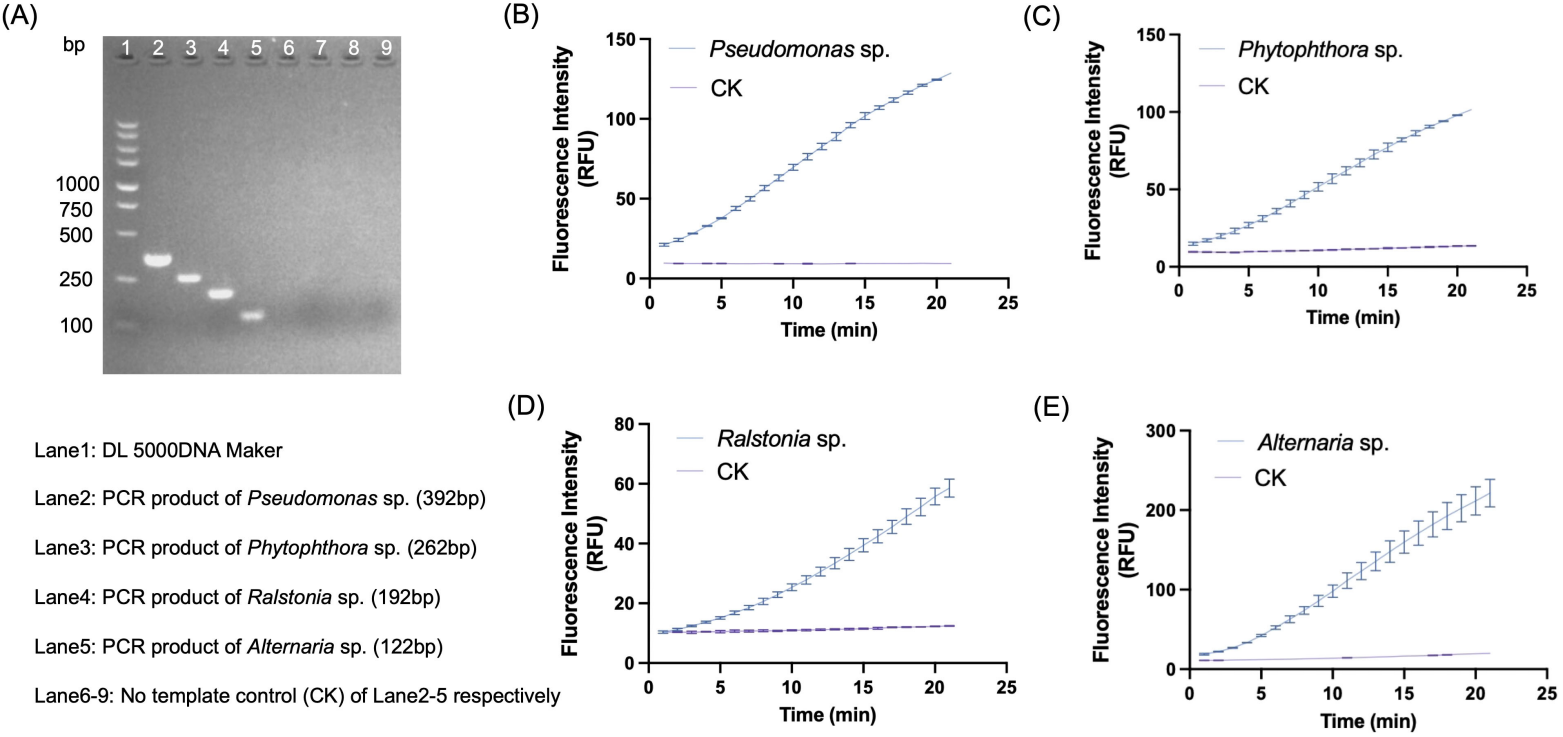
Feasibility verification of the singleplex PCR–Cas12a detection system for four pathogens. **(A)** Agarose gel electrophoresis results of singleplex PCR amplification. **(B-E)** Fluorescence detection curve in the singleplex PCR–Cas12a system of *Pseudomonas* sp., *Phytophthora* sp., *Ralstonia* sp., and *Alternaria* sp., respectively. CK indicates a control in which no template was present. Error bars represent the standard deviation of three independent replicates.

As shown in **Figure 2A**, distinct single bands of the expected sizes were observed for all four targets (lanes 2–5), confirming successful and specific amplification without detectable nonspecific by-products. No amplification was observed in the no-template controls (lanes 6–9), indicating the high specificity of the designed primers.

Subsequently, each individual PCR product was subjected to a singleplex Cas12a reaction containing the corresponding crRNA, and fluorescence signals were recorded over time. As illustrated in **Figures 2B–E**, all four Cas12a assays generated steadily increasing fluorescence signals in the presence of their specific targets, while the no-template controls showed no signal increase. These results collectively demonstrate that each primer–crRNA pair is both feasible and highly specific for its corresponding pathogen.

**Figure 3:**
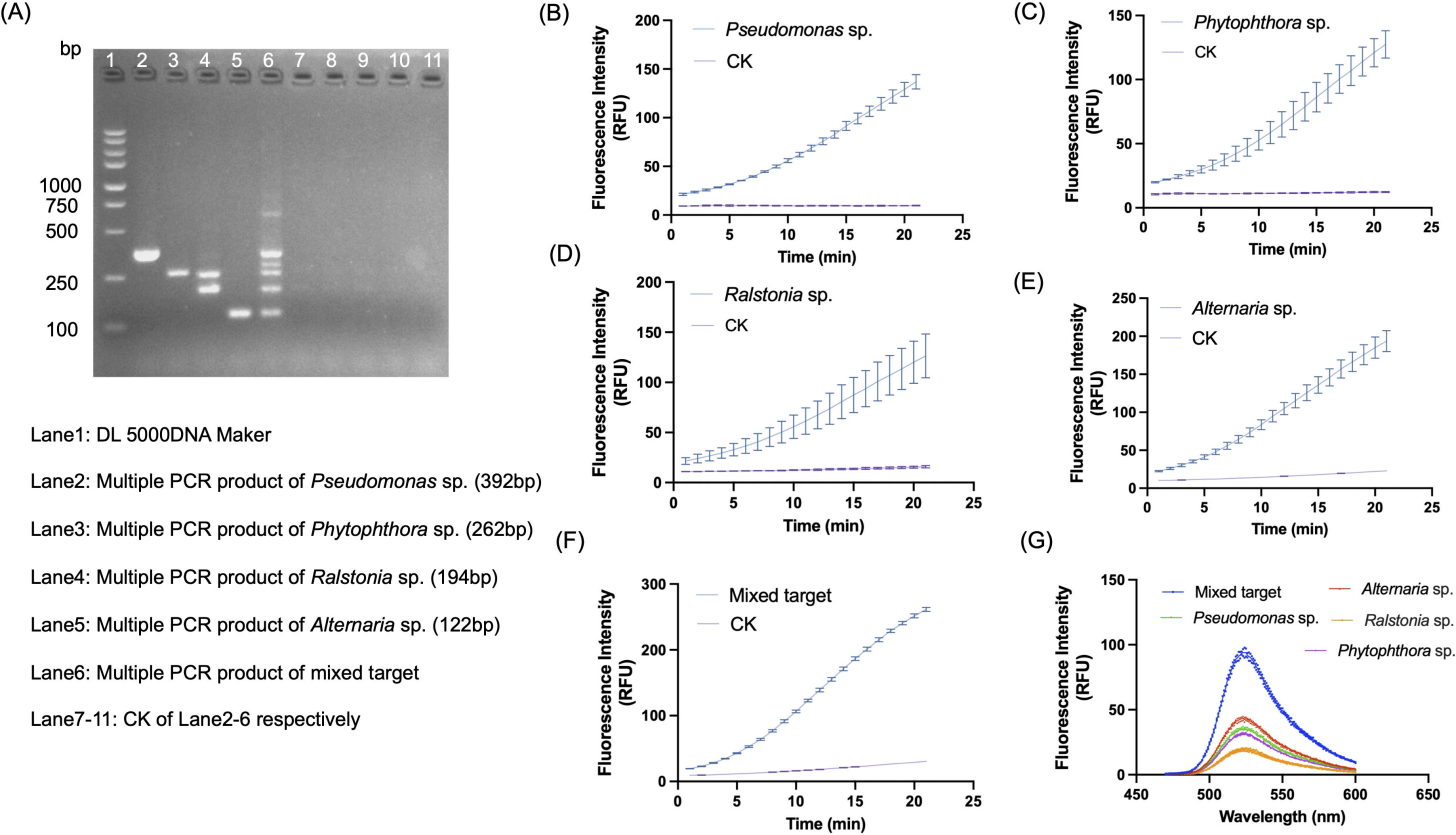
Feasibility verification of the multiplex PCR–Cas12a detection system for four pathogens. **(A)** Agarose gel electrophoresis results of multiplex PCR amplification. **(B-F)** Fluorescence detection curve in the singleplex PCR–Cas12a system of *Pseudomonas* sp., *Phytophthora* sp., *Ralstonia* sp., *Alternaria* sp., and mixed pathogen target, respectively. **(G)** Fluorescence spectra for the detection of four individual pathogen targets and the mixed pathogen target. CK indicates a control in which no template was present. Error bars represent the standard deviation of three independent replicates.

To further evaluate the feasibility of multiplex PCR amplification system, the four primer sets targeting *Ralstonia* sp., *Phytophthora* sp., *Alternaria* sp., and *Pseudomonas* sp. were combined into a single reaction system. Artificially synthesized DNA fragments, representing each pathogen either individually or in combination, were used as templates. As shown in **Figure 3A**, when a single target template was present (lanes 2–5), clear and distinct bands were obtained for *Phytophthora* sp., *Alternaria* sp., and *Pseudomonas* sp. (lanes 2, 3, and 5), whereas two bands were detected for *Ralstonia* sp. (lane 4). The presence of multiple bands suggests partial nonspecific amplification, likely resulting from primer–primer interactions within the multiplex system. When all four templates were combined (lane 6), six bands were observed, further indicating potential cross-reactivity and mutual interference among primer pairs.

To verify whether these multiplex PCR products could still be effectively recognized by the Cas12a system, the resulting amplicons were introduced into multiplex Cas12a reactions, and fluorescence kinetics were monitored. As shown in **Figures 3B–E**, reactions containing single templates exhibited progressively increasing fluorescence signals, whereas the no-template controls remained unchanged. Similarly, when mixed templates were used (**Figures 3F, G**), distinct fluorescence signals were clearly observed, well separated from background levels in the controls.

Collectively, these findings confirm that the integrated multiplex PCR–Cas12a detection platform is both technically feasible and functionally reliable for simultaneous detection of single or multiple target pathogens. This combined system demonstrates strong potential for rapid, accurate, and high-throughput screening of complex disease samples in agricultural diagnostics.

### 3.3 Optimization of reaction conditions

#### 3.3.1 Optimization of the multiplex PCR reaction

In multiplex PCR assays, primer–primer interactions represent a key factor affecting amplification efficiency and specificity. To ensure robust performance of the developed multiplex PCR–Cas12a system, systematic optimization was performed to minimize primer cross-reactivity and achieve balanced amplification among all targets.

**Figure 4:**
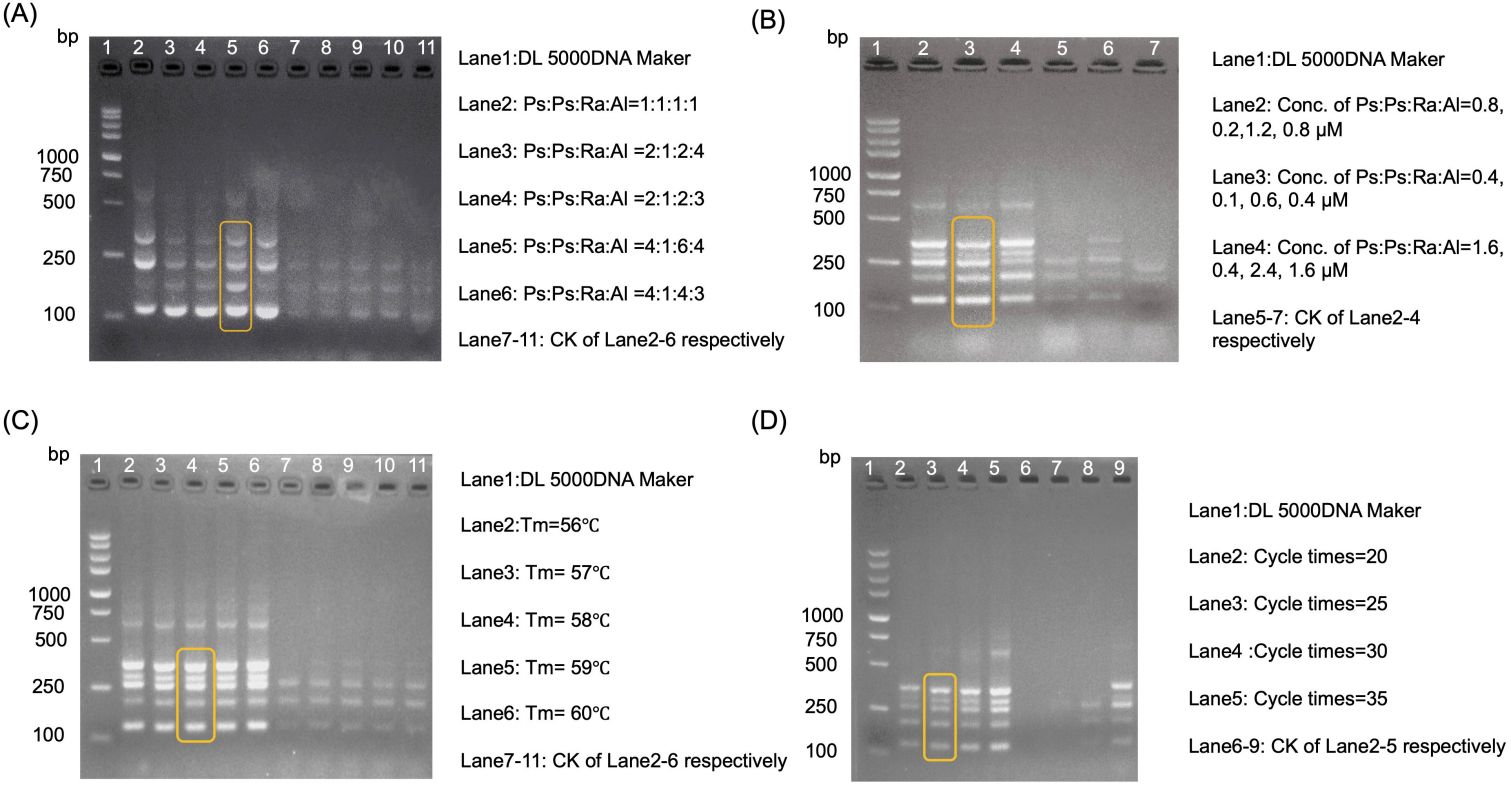
Optimization of the multiplex PCR reaction. **(A)** Primer ratio. **(B)** Primer concentration. **(C)** Annealing temperature. **(D)** Cycle number. The yellow boxes indicate the optimized conditions. CK indicates a control in which no template was present.

The relative proportions and concentrations of primers were first optimized. As shown in **Figure 4A**, when the primer ratio for *Pseudomonas* sp., *Phytophthora* sp., *Ralstonia* sp., and *Alternaria* sp. was adjusted to 4:1:6:4, all four target bands appeared uniformly bright and well defined (lane 5), indicating comparable amplification efficiencies across targets under these conditions. Building upon this ratio, primer concentrations were further optimized (**Figure 4B**). The results showed that when the final primer concentrations were 0.4, 0.1, 0.6, and 0.4 μM, respectively, nonspecific amplification was markedly reduced (lane 3), suggesting minimal primer interference. Therefore, this combination of primer ratio (4:1:6:4) and concentration (0.4, 0.1, 0.6, and 0.4 μM) was selected as optimal for subsequent assays.

Next, the annealing temperature and cycle number were optimized to further enhance amplification specificity. As shown in **Figure 4C**, varying the annealing temperature produced only minor changes in amplification intensity. An annealing temperature of 58 °C was ultimately chosen, as it yielded strong target bands while maintaining low nonspecific amplification.

Optimization of the PCR cycle number (**Figure 4D**) revealed that nonspecific amplification in no-template controls increased progressively with higher cycle counts. When the number of cycles was set to 20 or 25, no visible nonspecific bands appeared in the negative controls. Considering both target band brightness and background suppression, 25 cycles were determined to be optimal for the multiplex PCR reaction.

Collectively, these optimizations—encompassing primer ratios, concentrations, annealing temperature, and cycle number—significantly improved amplification balance and specificity, establishing reliable reaction parameters for the multiplex PCR–Cas12a detection platform.

#### 3.3.2 Optimization of the Cas12a reaction

To further enhance the performance of the multiplex PCR–Cas12a assay, the concentrations of the four pathogen-specific crRNAs were optimized to achieve balanced detection efficiency across all targets. The objective was to ensure comparable fluorescence responses for each pathogen when multiplex PCR products were used as templates in single-crRNA Cas12a assays.

**Figure 5:**
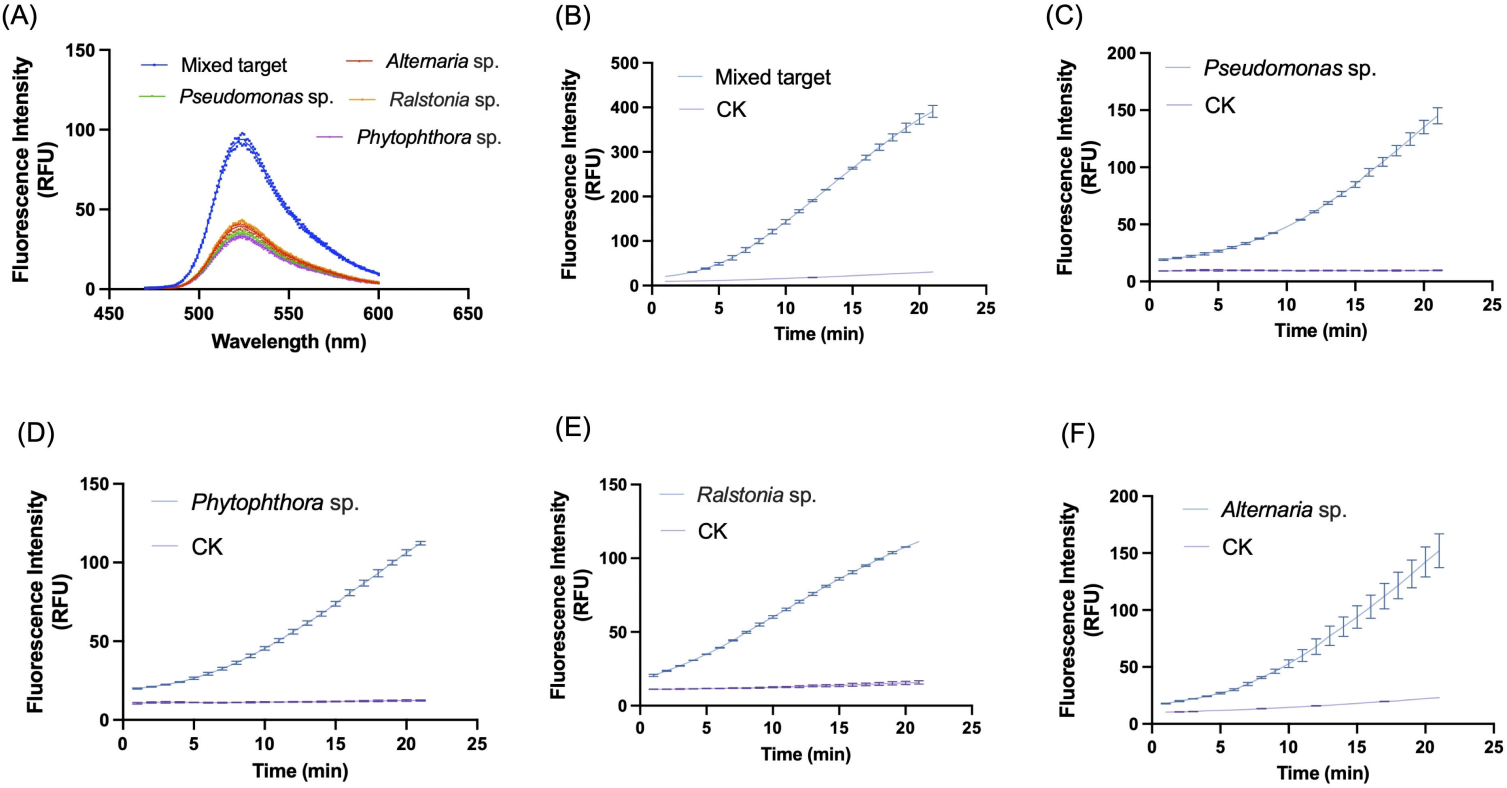
Optimization of the Cas12a reaction. **(A)** Fluorescence spectra after crRNA ratio optimization for the detection of four individual pathogen targets and the mixed pathogen targets. **(B–F)** Fluorescence detection curves in the optimized multiplex PCR–Cas12a system for the mixed pathogen targets, *Pseudomonas* sp., *Phytophthora* sp., *Ralstonia* sp., and *Alternaria* sp., respectively. CK indicates a control in which no template was present. Error bars represent the standard deviation of three independent replicates.

As shown in **Figure 5A**, after optimization, the fluorescence intensities of the four pathogen targets were maintained within the range of 100–150 arbitrary units, reflecting well-balanced detection sensitivity. The optimal crRNA concentrations for *Pseudomonas* sp., *Phytophthora* sp., *Ralstonia* sp., and *Alternaria* sp. were determined to be 60, 60, 60, and 30 nM, respectively (**Figures 5C–F**).

Compared with the pre-optimization results (**Figure 3G**), the optimized system produced more uniform fluorescence profiles across all targets (**Figure 5A**), indicating that signal disparities caused by differential crRNA activities had been effectively minimized. Compared with the pre-optimization results, the optimized conditions reduced the differences in fluorescence output among the four target groups. However, the target-specific LOD results show that the four primer–amplicon–crRNA combinations were not analytically equivalent. Thus, the optimization improved overall signal balance but did not eliminate target-dependent amplification and Cas12a-recognition bias.

### 3.4 Sensitivity, specificity, and visual detection

**Figure 6:**
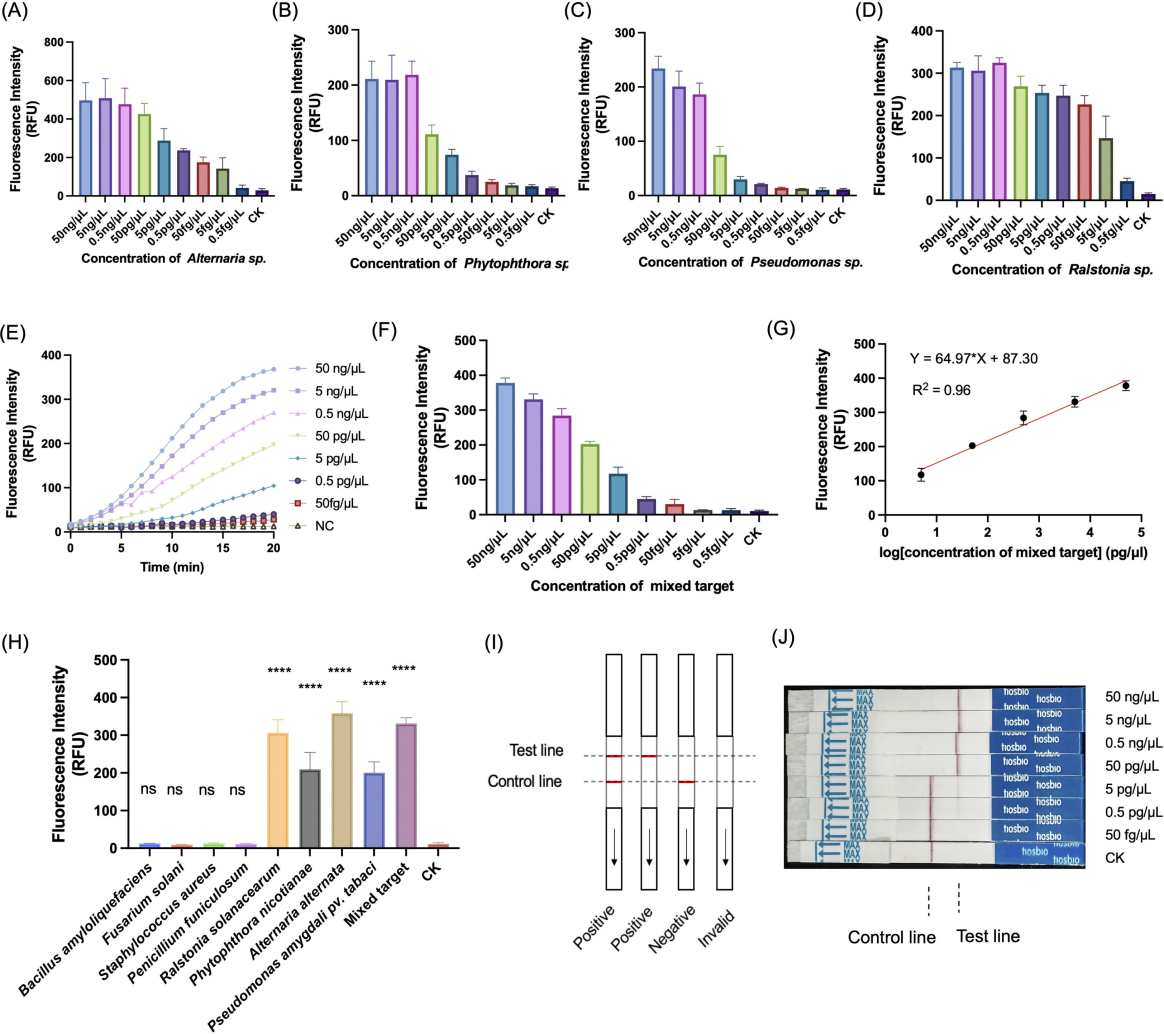
Sensitivity, specificity, and visual detection of the proposed multiplex PCR–Cas12a detection method. **(A–D)** Sensitivity analysis for *Alternaria* sp., *Phytophthora* sp., *Pseudomonas* sp., and *Ralstonia* sp. when used as individual targets. **(E)** Time-dependent fluorescence amplification curves for the mixed pathogen targets. **(F)** Sensitivity analysis of the multiplex PCR–Cas12a system for the mixed pathogen targets. **(G)** Linear range of the multiplex PCR–Cas12a system for the mixed pathogen targets. **(H)** Specificity analysis of the multiplex PCR–Cas12a detection method. “ns” represents not significant (p>0.05), and “****” represents a highly significant difference (p<0.0001). **(I)** Schematic illustration of the visual detection process based on the combination of the multiplex PCR–Cas12a system with LFD. **(J)** Sensitivity of the multiplex PCR–Cas12a–LFD detection method for mixed pathogen targets. CK indicates a control in which no template was present. Error bars represent the standard deviation of three independent replicates.

To determine the analytical sensitivity of the multiplex PCR–Cas12a detection platform, artificially synthesized DNA fragments from *Alternaria* sp., *Phytophthora* sp., *Pseudomonas* sp., and *Ralstonia* sp. were tested at concentrations ranging from 50 ag/μL to 50 ng/μL. Fluorescence signal changes were recorded and analyzed (**Figures 6A–D**). The target-specific mass-based LODs for the *Alternaria*, *Phytophthora*, *Pseudomonas*, and *Ralstonia* target groups were 5 fg/μL, 0.5 pg/μL, 5 pg/μL, and 5 fg/μL, respectively. The exact plasmid lengths and corresponding copy-number equivalents (ranging from 1.06×10^3^ to 1.29×10^6^ copies/μL) are detailed in **Supplementary Table 5**. The four target groups exhibited different fluorescence profiles and target-specific analytical sensitivities. These differences likely reflect the combined effects of target molar copy number at a given mass concentration, primer-dependent amplification efficiency in the multiplex PCR, amplicon sequence characteristics, crRNA–target hybridization, PAM/protospacer context, and Cas12a activation kinetics. Although the primer and crRNA concentrations were optimized to improve response balance, this optimization did not make the four target-specific reactions analytically identical. Under the tested conditions, the *Pseudomonas* target group showed the highest mass-based LOD and was therefore the least sensitive individual target, whereas the *Alternaria* and *Ralstonia* target groups were detectable at lower target mass concentrations.

To further assess the assay’s performance in the presence of multiple targets, the four DNA templates were mixed at equal proportions and analyzed under the same conditions (**Figures 6E–G**). The fluorescence intensity exhibited a strong linear correlation with the logarithm of mixed target concentrations within the range of 5 pg/μL to 50 ng/μL (**Figure 6G**). The fitted regression equation, Y = 64.97 × X + 87.30 (R^2^ = 0.96), demonstrated excellent linearity, where *X* represents the logarithm of the mixed target concentration and *Y* the relative fluorescence intensity of the assay. Using the formula LOD = 3 × SD/slope—where *SD* denotes the standard deviation of the negative control and *slope* is derived from the fitted curve—the calculated detection limit was 186 fg/μL (equivalent to a combined total of 4.44×10^4^ target/μL; **Supplementary Table 5**), confirming that the system maintains high sensitivity even under multiplex conditions.

The preliminary analytical exclusivity of the assay was evaluated using the four target templates, their mixture, and three selected non-target templates. As shown in **Figure 6H**, detectable fluorescence signals were obtained from the four target groups and the mixed-target sample, whereas the tested non-target templates produced signals comparable to the no-template control. These results indicate that no detectable cross-reactivity occurred within the limited experimental panel examined in this study. The Cas12a step provides an additional sequence-dependent recognition layer after multiplex PCR because reporter cleavage requires an amplicon containing the appropriate PAM-adjacent protospacer. This design can reduce the likelihood that primer dimers or unrelated nonspecific amplicons generate a positive readout. However, it does not eliminate all possible cross-reactivity, particularly when a closely related organism contains both compatible primer-binding sites and the corresponding Cas12a target sequence. Therefore, the present results should not be interpreted as comprehensive exclusivity against all tobacco-associated microorganisms or as evidence of pathogenic-strain specificity.

To enable rapid and user-friendly visual detection, the fluorophore–quencher (F–Q) probe was replaced with a fluorophore–biotin (F–B) probe for lateral flow dipstick (LFD) readout. As shown in **Figure 6I, J**, distinct test lines (T-lines) appeared in the presence of mixed target samples, with a visual detection limit of 0.5 pg/μL. Collectively, these findings confirm that the multiplex PCR–Cas12a–LFD assay provides a rapid, highly sensitive, and visually interpretable diagnostic platform capable of pooled multi-target screening of multiple tobacco pathogens without the need for sophisticated instrumentation, offering a practical solution for rapid pathogen surveillance and early disease management. Compared to previously reported CRISPR-based detection methods, this assay demonstrates comparability in terms of assay time, workflow complexity, detection limits, and cost; moreover, it strikes a balance by enabling the screening of multiple targets through a relatively convenient and cost-effective approach (**Supplementary Table 4**).

While the platform demonstrated high sensitivity and practical utility as a primary alert system, certain inherent taxonomic limitations must be acknowledged. The diagnostic targets employed—specifically the 16S rRNA and ITS regions—are robust and highly conserved for broad detection; however, they may lack the hyper-resolution necessary for discriminating at the pathovar level (e.g., *Pseudomonas syringae pv. tabaci*). Therefore, a positive result from this assay should be clinically interpreted as an alert for the genus or species-complex level of the suspect pathogens. For definitive agricultural quarantine or precise epidemiological tracing that requires pathovar differentiation, subsequent whole-genome sequencing or pathovar-specific assays would still be required. Nonetheless, for rapid, syndromic pre-transplantation screening or early symptomatic testing, this genus/species-complex level resolution provides a critical temporal advantage for disease management.

### 3.5 Detection in spiked samples

**Figure 7:**
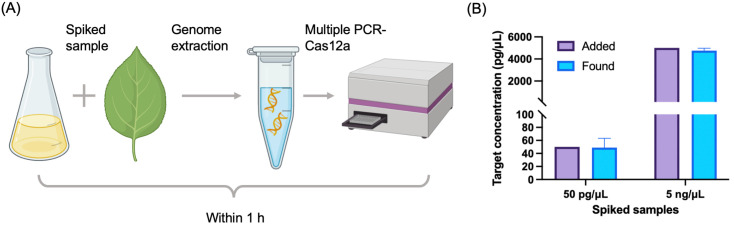
Recovery test in spiked samples. **(A)** Workflow of the detection procedure. **(B)** Recovery results of spiked samples analyzed using the multiplex PCR–Cas12a detection method. Error bars represent the standard deviation of three independent replicates.

To preliminarily evaluate the compatibility of the multiplex PCR–Cas12a assay with a tobacco-leaf matrix, simulated mixed-pathogen samples were prepared using homogenized, apparently disease-free tobacco leaves and analyzed under the optimized conditions (**Figure 7**, **Supplementary Figure S5**). At the two tested extracted-DNA levels of 50 pg/μL and 5 ng/μL, the apparent recovery rates were 93% and 95%, respectively. These results indicate that the assay retained acceptable analytical performance in the tested spiked-leaf preparations.

However, these recovery results should not be interpreted as a pathogen burden–based limit of detection in tobacco leaves. The microbial materials used for spiking were not independently enumerated before sample preparation, and the amount of host DNA was not systematically varied against a fixed low pathogen concentration. Therefore, the current data cannot establish a minimum detectable burden expressed as CFU/g, cells/g, spores/g, or propagules/g, nor can they demonstrate that increasing amounts of plant nucleic acid have no effect on the detection of very low pathogen burdens. Instead, the results provide preliminary evidence of matrix compatibility only at the two tested extracted-DNA levels.

It should be noted that the current multiplex PCR-Cas12a-LFD workflow involves multiple discrete steps and relies on thermal cycling equipment, which restricts its application for true point-of-care or on-site field testing. However, given the inherent limitations of fully isothermal specific primer multiplexing (e.g., severe primer interference), balancing operational simplicity with the capacity for multi-target concurrent screening remains a challenge. Therefore, this multi-step design was adopted as a necessary trade-off to ensure high-fidelity multiplex screening in laboratory settings.

While the current platform has demonstrated exceptional analytical sensitivity and robust resistance to matrix interference in spiked whole-leaf homogenates, we acknowledge certain limitations in this methodological proof-of-concept study. Specifically, the validation primarily relied on artificially synthesized targets and spiked healthy plant matrices to establish standardized quantitative metrics (e.g., LOD and recovery rates). Naturally infected field samples exhibit highly variable pathogen loads, complex co-infection dynamics, and varying degrees of tissue necrosis, which may further challenge nucleic acid extraction and amplification efficiency. Therefore, prior to broad deployment in agricultural settings, large-scale field studies utilizing naturally infected cohorts are necessary to fully evaluate the field diagnostic performance and practical reliability of this multiplex system.

## 4 Discussion

Rapid and reliable detection of tobacco-associated pathogens is important for early disease warning, sample triage, and timely disease-management decisions. Tobacco bacterial wilt, black shank, brown spot, and wildfire may cause overlapping or nonspecific symptoms during early disease development, while their associated microorganisms may also occur in soil, irrigation water, seedbeds, and plant tissues before severe symptoms become evident. Consequently, nucleic-acid-based screening can complement visual assessment by providing sequence-dependent evidence of the presence of relevant microbial target groups. In this study, we developed a multiplex PCR–Cas12a platform with fluorescence and LFD readouts for pooled screening of four target groups associated with these major tobacco diseases.

CRISPR/Cas12a has been increasingly incorporated into nucleic-acid diagnostic platforms because target-dependent activation of its collateral ssDNA cleavage activity permits sensitive fluorescence or lateral-flow reporting (Huang et al., 2025). Recent studies have further explored multiplex amplification–Cas12a strategies for human pathogens, and genetically modified crops, including multiplex RPA–Cas12a and LAMP–Cas12a configurations (Ding et al., 2025; Li et al., 2025b; Zhou et al., 2025),. These studies demonstrate the potential of CRISPR-based multiplex screening but also highlight the trade-offs among primer complexity, target differentiation, workflow integration, instrumentation, and cost. In plant-pathogen diagnostics, isothermal amplification and LFD approaches offer operational advantages, particularly for single-target detection (Wang et al., 2025a; Yue et al., 2026). However, combining several primer sets in one amplification reaction may increase competition, primer–dimer formation, and nonspecific amplification. The present study therefore used multiplex PCR as a practical laboratory-based amplification strategy to support pooled screening of four target groups.

This work builds on our previously reported PCR–Cas12a–LFD approach for detecting the same four tobacco-associated target groups (Niu et al., 2026). In the previous work, the target groups were evaluated using separate pathogen-specific PCR–Cas12a–LFD configurations, whereas the present study combines four primer pairs in a single multiplex PCR and four target-recognition crRNAs in a pooled Cas12a reaction. The principal methodological advance is therefore not the first use of PCR–Cas12a–LFD for these organisms, but the development and optimization of a pooled multi-target screening workflow. Specifically, primer ratios, primer concentrations, annealing temperature, cycle number, and crRNA concentrations were adjusted to reduce amplification imbalance and improve the comparability of responses among the target groups. At the same time, because the final fluorescence and LFD outputs use a universal ssDNA reporter, the current assay provides a collective positive/negative alert rather than four independently resolved pathogen identities.

The optimization results illustrate the importance of controlling amplification competition in a multiplex PCR system. Before optimization, additional bands were observed for some target and mixed-template reactions, indicating primer interactions or nonspecific amplification. Adjustment of the primer ratio and concentration, together with selection of 58 °C and 25 PCR cycles, improved amplification balance and reduced detectable background products. The subsequent Cas12a step provides an additional sequence-dependent recognition layer because reporter cleavage requires an amplicon containing the appropriate PAM-adjacent protospacer. This design can reduce the likelihood that primer dimers or unrelated nonspecific amplicons generate a positive readout. However, Cas12a recognition should not be interpreted as eliminating all false-positive risks. A nontarget or closely related organism containing both compatible primer-binding sites and the corresponding Cas12a target sequence could still activate the reporter. The Cas12a step therefore increases analytical selectivity relative to PCR readout alone but does not replace comprehensive exclusivity validation.

The four target groups showed different single-target analytical sensitivities, with mass-based LODs ranging from 5 fg/μL to 5 pg/μL under the tested conditions. Such differences may result from combined variation in primer amplification efficiency, amplicon length and sequence composition, template accessibility, crRNA–target hybridization, protospacer context, and Cas12a activation kinetics. Although crRNA concentrations were adjusted to reduce signal imbalance, these results do not demonstrate identical recognition efficiency for all four targets. In particular, the higher mass-based LOD observed for the *Pseudomonas* target group indicates that it was the least sensitive of the four single-target assays under the evaluated conditions.

For the equal-proportion mixed-template series, the calculated value of 186 fg/μL represents the analytical threshold at which the pooled universal reporter signal could be distinguished from the negative background. It should not be interpreted as a pathogen-specific LOD applicable independently to all four target groups. Because any sufficiently active target can contribute to the common fluorescence signal, stronger-performing targets may dominate the pooled response and may mask weaker amplification or recognition of another target. Therefore, the 186 fg/μL pooled-signal LOD does not guarantee detection of the least sensitive target, particularly the *Pseudomonas* target group, when that target is present alone or at a low relative abundance in a mixed sample. The target-specific LOD values remain more appropriate for interpreting the sensitivity of each individual target group. Moreover, the current mixed-template experiment used equal target proportions and does not fully reproduce the highly unequal pathogen burdens that may occur during natural coinfection.

The current use of a universal reporter is an important limitation in interpreting the platform as a multiplex assay. The multiplex PCR and pooled crRNA reaction allow four target groups to be screened concurrently in a shared workflow, but the final signal only indicates that at least one target group is present. It does not determine which target generated the signal, whether more than one target is present, or the relative abundance of the targets. Accordingly, the method is most accurately described as a pooled multi-target screening platform rather than a pathogen-resolved multiplex identification system. If target identity is required, the positive sample must be subjected to downstream pathogen-specific reactions, sequencing, culture-based identification, or another validated confirmatory assay. Future pathogen-resolved formats could incorporate reaction partitioning, spatially separated LFD test lines, orthogonal Cas enzymes, distinguishable fluorescent reporters, barcoded reporters, or microfluidic compartmentalization.

Preliminary spiked-leaf testing suggested that the assay is compatible with tobacco-derived sample matrices under the evaluated conditions. Tobacco tissues contain polysaccharides, polyphenols, secondary metabolites, and abundant host nucleic acids that may inhibit PCR amplification or Cas12a-mediated detection. Apparent recovery rates of 93%–95% were obtained at the two tested extracted-DNA levels, indicating that severe inhibition was not observed under these specific experimental conditions. Nevertheless, the host DNA background was not independently increased while maintaining a fixed low pathogen concentration. The present experiment therefore does not establish that high concentrations of plant nucleic acid have no effect on the detection of low-abundance pathogens.

Furthermore, the analytical LOD values determined using purified target nucleic acids cannot be directly converted into CFU/g, cells/g, spores/g, or propagules/g of leaf material. Such conversion depends on multiple pathogen- and sample-specific factors, including genome size, 16S rRNA or ITS copy number, growth stage, viability, the proportion of fungal or oomycete mycelium and reproductive structures, pathogen distribution within the tissue, DNA extraction efficiency, and the abundance of host-derived inhibitors. Because the pathogen inocula in the present study were not independently enumerated before spiking, a biological detection threshold in tobacco leaves could not be determined. Thus, the current findings should be regarded as analytical proof-of-concept and preliminary matrix-compatibility data rather than a fully established leaf-level diagnostic detection limit.

The present assay was designed as a broad target-group screening platform rather than a pathogenic-strain or pathovar-level confirmatory assay. The selected 16S rRNA and ITS regions provide broad taxonomic coverage but are not virulence markers and may be shared by pathogenic and non-pathogenic members of the same or closely related taxa. For example, the in silico analysis indicates that the *Pseudomonas* primer pair may cover not only *P. syringae* and *P. syringae* pv. *tabaci* but also other *Pseudomonas* taxa, including *P. fluorescens* and *P. putida*. Consequently, a positive signal should be interpreted as detection of DNA belonging to at least one target taxonomic group rather than definitive evidence of a specific pathogenic strain.

In addition, nucleic-acid detection does not independently establish organism viability, active infection, or causal association with observed disease symptoms. The Cas12a recognition step improves sequence-dependent analytical selectivity by requiring the appropriate PAM-adjacent protospacer, but it cannot determine pathogenic phenotype when pathogenic and non-pathogenic organisms share the same target sequence. Therefore, definitive diagnosis, epidemiological tracing, quarantine confirmation, or pathovar differentiation would require complementary evidence from culture-based identification, sequencing, pathogenicity-associated markers, or validated pathovar-specific molecular assays. Despite this limitation, target-group-level screening can still provide an early warning that supports sample prioritization and subsequent confirmatory analysis.

The experimental specificity panel also represents a preliminary rather than comprehensive exclusivity assessment. No detectable cross-reactivity was observed among the limited non-target templates tested, but this result cannot exclude possible reactions with all closely related taxa, tobacco-associated microbiota, or other plant pathogens. Similarly, the current validation relied mainly on synthesized target sequences and spiked tobacco-leaf preparations rather than naturally infected field samples. Naturally infected tissues may contain unevenly distributed pathogens, low target abundance, degraded nucleic acids, mixed microbial communities, variable host DNA backgrounds, and tissue-derived inhibitors. These factors may affect performance differently from standardized spiked samples. Consequently, the present study establishes analytical feasibility but does not yet provide estimates of field diagnostic sensitivity, field diagnostic specificity, positive predictive value, or negative predictive value.

The current workflow also requires DNA extraction, thermal cycling, transfer of PCR products, separate Cas12a incubation, and subsequent fluorescence or LFD readout. It is therefore more appropriate for centralized laboratories or plant-protection service stations than for fully equipment-free field testing. Future development should focus on simplified sample preparation, reduced reaction volumes, closed-tube amplification and Cas12a detection, lyophilized reagents, contamination control, and pathogen-resolved output. Validation using independently quantified microbial inocula and naturally infected samples from different tobacco-growing regions will also be necessary before routine agricultural deployment.

In conclusion, the present study demonstrates the feasibility of combining multiplex PCR with pooled Cas12a recognition and fluorescence or LFD reporting for broad screening of four tobacco-associated pathogen groups. The platform provides a common alert when at least one target group is detected and introduces a second sequence-dependent recognition step after multiplex amplification. However, the current universal-reporter format does not differentiate individual targets, and its pooled-signal LOD should not be interpreted as the target-specific sensitivity of every pathogen group. Within these defined boundaries, the method represents a laboratory-based analytical proof of concept with potential value for preliminary sample screening and prioritization for confirmatory testing.

## Data Availability

The original contributions presented in the study are included in the article/**Supplementary Material**. Further inquiries can be directed to the corresponding author/s.

## Supplementary material

Supplementary Table 1 Specific DNA primers, crRNA sequences and probes.

Supplementary Table 2 In silico taxonomic inclusivity of the primer pairs within selected target taxa.

Supplementary Table 3 Preliminary in silico comparison of the primer pairs against three selected non-target genera.

Supplementary Table 4 Comparison of the developed detection method with previously reported methods.

Supplementary Table 5 Calculation of copy-number equivalents for the single and mixed templates.

Supplementary Figure 1 In silico taxonomic inclusivity of the *Ralstonia* sp.-F/R primer pair.

Supplementary Figure 2 In silico taxonomic inclusivity of the *Phytophthora* sp.-F/R primer pair.

Supplementary Figure 3 In silico taxonomic inclusivity of the *Alternaria* sp.-F/R primer pair.

Supplementary Figure 4 In silico taxonomic inclusivity of the *Pseudomonas* sp.-F/R primer pair.

Supplementary Figure 5 Representative lateral flow dipstick results for the spiked-leaf samples.
