# Supplementary material for: A multiplex PCR–Cas12a–lateral flow platform for pooled screening of four major tobacco pathogen groups
Source: Front Plant Sci. 2026 Aug 4;17:1919480. doi: 10.3389/fpls.2026.1919480 (PMC13483075; doi:10.3389/fpls.2026.1919480)
Supplement: Supplementary file 1 [file DataSheet1.docx]

**A Multiplex PCR–Cas12a–Lateral Flow Platform for Pooled Screening of Four Major Tobacco Pathogen Groups**

Yena Zhang^1, 2^, Changwen Ye^1^, Bo Zeng^1^, Hongru Xi^1^, Baojian Wu^1^, Kuo Huang^1^, Dandan Liu^1^, Guokang Chen^2^, Chenqi Niu^1, *^

^1^China Tobacco Standardization Research Center, Zhengzhou Tobacco Research Institute of CNTC,

Zhengzhou, China, 450000.

^2^College of Plant Protection, Southwest University, Chongqing, China, 400715.

Corresponding author

**Chenqi Niu**

Email: niucq_ztri@163.com

Zhengzhou Tobacco Research Institute of CNTC, China Tobacco Standardization Research Center

Zhengzhou, Henan, China, 450000

1. **Tables**

Table S1. Specific DNA primers, crRNA sequences and probes

| Name | Target locus | Sequences (5’-3’) |
| --- | --- | --- |
| *Ralstonia* sp.-F | 16S rRNA | TCTGAGAGGACGATCAGCCA |
| *Ralstonia* sp.-R |  | TACCGTCATCCACTCCAGGT |
| *Ralstonia* sp.-crRNA |  | UAAUUUCUACUAAGUGUAGAUGUCCGGAAAGAAAUCGCUUC |
| *Phytophthora* sp.-F | ITS | TTGCACTTCCGGGTTAGTCC |
| *Phytophthora* sp.-R |  | ACTCTTCCATTAACGCCGCA |
| *Phytophthora* sp.-crRNA |  | UAAUUUCUACUAAGUGUAGAUCUCGAAAAGUGGUGGCGUUG |
| *Alternaria* sp.-F | ITS | TGAAGAACGCAGCGAAATGC |
| *Alternaria* sp.-R |  | CAAATGACGCTCGAACAGGC |
| *Alternaria* sp.-crRNA |  | UAAUUUCUACUAAGUGUAGAUAACGCACAUUGCGCCCUUUG |
| *Pseudomonas* sp -F | 16S rRNA | AACGGACGCTAATACCGCAT |
| *Pseudomonas* sp -R |  | AACGCTTGCACCCTCTGTAT |
| *Pseudomonas* sp -crRNA |  | UAAUUUCUACUAAGUGUAGAUGACGUUACCGACAGAAUAAG |
| F-Q probe | - | FAM-TTATT-BHQ1 |
| F-B probe | - | FAM-TTATT-biotin |

FAM, 6-carboxyfluorescein; BHQ1, Black Hole Quencher 1; F–Q, fluorophore–quencher reporter; F–B, fluorophore–biotin reporter.

Table S2. In silico taxonomic inclusivity of the primer pairs within selected target taxa

|  | Taxon queried | Number of core_nt sequence records meeting the specified matching criteria |
| --- | --- | --- |
| *Ralstonia* sp.-F/R primer pair | *Ralstonia solanacearum* | 644 |
|  | *Ralstonia pseudosolanacearum* | 355 |
|  | *Ralstonia syzygii* | 66 |
| *Phytophthora* sp.-F/R primer pair | *Phytophthora nicotianae* | 896 |
|  | *Phytophthora capsici* | 46 |
|  | *Phytophthora cinnamomi* | 500 |
| *Alternaria* sp.-F/R primer pair | *Alternaria alternata* | 1000 |
|  | *Alternaria tenuissima* | 330 |
|  | *Alternaria solani* | 695 |
| *Pseudomonas* sp -F/R primer pair | *Pseudomonas syringae* | 739 |
|  | *Pseudomonas syringae pv. tabaci* | 21 |
|  | Pseudomonas fluorescens | 833 |
|  | Pseudomonas putida | 358 |

Counts represent NCBI core_nt sequence records rather than unique strains or experimentally verified pathogenic isolates. Sequence matching indicates potential taxonomic inclusivity and should not be interpreted as evidence of pathogenicity.

Table S3. Preliminary in silico comparison of the primer pairs against three selected non-target genera

| Number of core_nt records meeting the defined primer-pair matching criterion | Rhizobium sp. | Streptomyces sp. | Pectobacterium sp. |
| --- | --- | --- | --- |
| *Ralstonia* sp.-F/R primer pair | 0 | 0 | 0 |
| *Phytophthora* sp.-F/R primer pair | 0 | 0 | 0 |
| *Alternaria* sp.-F/R primer pair | 0 | 0 | 0 |
| *Pseudomonas* sp -F/R primer pair | 3 | 2 | 0 |

Table S4. Comparison of the Developed Detection Method with Previously Reported Methods

| Number | Technology Description | Detection target | Readout Format | Detection limit | Detection time | workflow complexity | Cost | Reference |
| --- | --- | --- | --- | --- | --- | --- | --- | --- |
| 1 | Microfluidic Device + Cas12a + Multiplex RPA | Multiplex | Fluorescence signal | 0.26 attomole/reaction | 40 min | High | High | Nat Commun., 2022, 13, 6480 |
| 2 | Cas12a+ Reporter G3 | Singleplex | Fluorescence signal +LFD | 50 pM | PCR+20min | Medium | Medium | Biosensors and Bioelectronics, 2021, 187, 113292 |
| 3 | Chip +Cas12a+RPA | Duplex | Fluorescence Imaging | 10 aM (6 copies /reaction) | 40 min | High | High | Analytical Chemistry, 2024, 96, 20602-20611 |
| 4 | PCR+HCR+Cas12a | Singleplex | Fluorescence signal | 20 CFU/ml | 105 min | High | High | Journal of Agricultural and Food Chemistry, 2024, 72(15): 8831-8839. |
| 5 | PCR+Cas12a | Singleplex | Fluorescence signal | 10 copies /reaction | 40 min | Medium | Medium | Front. Microbiol. 2023.14:1128261. |
| 6 | PCR+Cas12a | Singleplex | Fluorescence signal | 1000 copies /mL | 170 min | Medium | Medium | Analytical chemistry, 2023, 95(45): 16692-16700. |
| 7 | aRPA+HCR+Cas12a | Singleplex | Fluorescence signal | 5 CFU/ml | 6 h | High | High | Biosensors and Bioelectronics, 2026: 118689. |
| 8 | Multiplex-PCR+Cas12a+LFD | Multiplex | Fluorescence signal +LFD | 186 fg/μL ( 4.44×10^4^ target copies/μL) | PCR+20min | Medium | Medium | This study |

Table S5. Calculation of copy-number equivalents for the single and mixed templates

| Target group / Mixture | Plasmid complete length (bp) | Mass-based LOD | Copies/μL *^a^* |
| --- | --- | --- | --- |
| *Alternaria* target group | 3360 | 5 fg/μL | 1.36×10^3^ |
| *Ralstonia* target group | 4291 | 5 fg/μL | 1.06×10^3^ |
| *Phytophthora* target group | 4282 | 0.5 pg/μL | 1.07×10^5^ |
| *Pseudomonas* target group | 3553 | 5 pg/μL | 1.29×10^6^ |
| Mixed Template*^b^* | Differing lengths | 186 fg/μL | 4.44×10^4^ (Total copies) |

*^a^* Copy numbers were calculated using the equation *N*=(*m*×*N_A_*)/(10^15^×660×*L*), where *L* is the complete plasmid length including the 2800-bp vector backbone.

*^b^* The mixed template contained equal initial mass proportions of the four plasmids. The total copy number is the sum of the components.

2. **Figures**

Figure S1. In silico taxonomic inclusivity of the *Ralstonia* sp.-F/R primer pair.

Figure S2. In silico taxonomic inclusivity of the *Phytophthora* sp.-F/R primer pair.

Figure S3. In silico taxonomic inclusivity of the *Alternaria* sp.-F/R primer pair.

Figure S4. In silico taxonomic inclusivity of the *Pseudomonas* sp.-F/R primer pair.

Figure S5. Representative lateral flow dipstick results for the spiked-leaf samples.
